# Disruption-Based Multiobjective Equilibrium Optimization Algorithm

**DOI:** 10.1155/2020/8846250

**Published:** 2020-11-30

**Authors:** Hao Chen, Weikun Li, Weicheng Cui

**Affiliations:** ^1^Zhejiang University—Westlake University Joint Training, Zhejiang University, Hangzhou 310024, China; ^2^Key Laboratory of Coastal Environment and Resources Research of Zhejiang Province, Institute of Advanced Technology, Westlake Institute for Advanced Study, 18 Shilongshan Road, Hangzhou 310024, China; ^3^School of Engineering, Westlake University, 18 Shilongshan Road, Hangzhou 310024, China

## Abstract

Nature-inspired computing has attracted huge attention since its origin, especially in the field of multiobjective optimization. This paper proposes a disruption-based multiobjective equilibrium optimization algorithm (DMOEOA). A novel mutation operator named layered disruption method is integrated into the proposed algorithm with the aim of enhancing the exploration and exploitation abilities of DMOEOA. To demonstrate the advantages of the proposed algorithm, various benchmarks have been selected with five different multiobjective optimization algorithms. The test results indicate that DMOEOA does exhibit better performances in these problems with a better balance between convergence and distribution. In addition, the new proposed algorithm is applied to the structural optimization of an elastic truss with the other five existing multiobjective optimization algorithms. The obtained results demonstrate that DMOEOA is not only an algorithm with good performance for benchmark problems but is also expected to have a wide application in real-world engineering optimization problems.

## 1. Introduction

Conventional mathematical optimization methods have the disadvantage of getting trapped in local optima for nonlinear optimization problems. Moreover, such optimization algorithms are highly complex and specialized. Inspired by the idea of biological evolution in nature, metaheuristic optimization algorithms have attracted huge attention due to the advantages of local avoidance and easy implementation. Research on optimization algorithms achieves rapid development due to the emergence of metaheuristic optimization algorithms. Many nature-inspired optimization algorithms have been proposed in the past few decades, including particle swarm optimization (PSO) [1], ant colony optimization (ACO) [2], evolution strategies (ES) [3], genetic algorithm (GA) [4], artificial bee colony algorithm (ABC) [5], gravitational search algorithm (GSA) [6], bat algorithm (BA) [7], flower pollination algorithm (FPA) [8], grey wolf optimizer (GWO) [9], whale optimization algorithm (WOA) [10], disruption particle swarm optimization (DPSO) [11], and equilibrium optimization algorithm (EO) [12]. Most of them are used to handle single objective optimization problems.

However, there is usually more than one objective needs to be optimized in real-world optimization problems, which means the common characteristic of real problems is multiobjective. In contrast to single objective problem, a multiobjective problem takes several conflicting objectives into consideration simultaneously. Instead of a single optimal solution, there is usually a set of alternative trade-offs between the objectives called Pareto optimal solutions in a multiobjective optimization problem [13]. Traditional methods to handle multiobjective optimization problems sometimes cannot produce well-distributed solutions along the Pareto front, and it may have difficulty in finding Pareto optimal solutions in nonconvex regions [14]. In 1985, Schaffer et al. [15] proposed the vector evaluated genetic algorithm (Vega) and applied it to solve the optimization problem involving multiple objectives for the first time. Then, a series of initial multiobjective optimization algorithms based on Pareto optimal were proposed successively, such as multiple objective genetic algorithms (MOGA) [16], niched Pareto genetic algorithm (NPGA) [17], and a nondominated sorting genetic algorithm (NSGA) [18]. The characteristics of these multiobjective optimization algorithms are the individual selection method based on nondominant ranking and the population diversity maintaining strategy based on fitness sharing mechanism. Due to the effectiveness of nature-inspired optimization algorithms, research on multiobjective optimization algorithms has attracted lots of attention in the past few decades. Some classical multiobjective evolutionary algorithms have been proposed, including nondominated sorting genetic algorithm version 2 (NSGA-II) [19], region-based selection in evolutionary multiobjective optimization (PESA2) [20], improving strength-Pareto evolutionary algorithm (SPEA2) [21], a multiobjective evolutionary algorithm based on decomposition (MOEA/D) [22], multi objective particle swarm optimization (MOPSO) [23], and multiobjective simulated-annealing algorithm (MOSA) [24]. In recent years, various novel multiobjective optimization algorithms have been proposed such as multiobjective gravitational search algorithm (MOGSA) [25], grid-based evolutionary algorithm (GrEA) [26], multiobjective grey wolf algorithm (MOGWO) [27], multiobjective ant lion optimizer (MOALO) [28], and multiobjective whale optimization (MOWOA) [29].

On the basis of absorbing the excellent searching mechanism of the equilibrium optimization algorithm and a novel mutation operator proposed in this work, this paper presented a disruption-based multiobjective equilibrium optimization algorithm (DMOEOA), which is able to handle multiobjective optimization problems. The novel mutation operator named layered disruption method is first proposed in this work with the aim of enhancing the exploration and exploitation abilities of DMOEOA. In addition, according to the No Free Lunch theorem [30], one optimization algorithm cannot solve all optimization problems effectively. This theorem also provides researchers with opportunities and motivations to propose new multiobjective optimization algorithms.

In this paper, the basic concepts of multiobjective optimization problems and grid mechanism are given in Section 2. The introduction of equilibrium optimization operator and layered disruption method is presented in Section 3. Section 4 provides experimental results and analysis of DMOEOA on benchmark functions with five multiobjective optimization algorithms. The analysis of the layered disruption method and the parametric study is also conducted in this section. In addition, the application of DMOEOA in the structural optimization of an elastic truss is presented in Section 5. Finally, some concluding remarks are given in Section 6.

## 2. Basic Concepts

In this section, the concepts of multiobjective optimization problems (MOPs) are given first, then some definitions of grid mechanism are provided.

### 2.1. Multiobjective Optimization Problems

The optimization of a problem with more than one objective is called multiobjective optimization. Without a loss of generality, the MOP can be formulated as a minimization problem as follows:

(1) $$\begin{array}{c} \text{Min}:F\left(X\right)={\left[f_{1}\left(X\right),f_{2}\left(X\right),\ldots ,f_{k}\left(X\right)\right]}^{T},\\ \text{S}.\text{t}.\;L_{s}\leq x_{s}\leq J_{s},\;s=1,2,\ldots ,n, \end{array}$$

where *X*=(*x*_1_, *x*_2_,…, *x*_*n*_) refers to the decision vector in the search space *R*^*n*^, *f*_*k*_(*X*) denotes the *k*^th^ objective to be optimized in the objective space *R*^*k*^, *L*_*s*_, and *J*_*s*_ represent the lower limit and upper limit of the decision variable, respectively.

**Definition 1 .**

(Pareto dominance). Given two decision vectors *X*, *Y*, the corresponding objective vectors are denoted as *F*(*X*), *F*(*Y*), respectively. *X* dominates *Y* (denoted as *X*≺*Y*) iff

(2) $$\begin{array}{c} \forall i\in \left(1,2,\ldots ,k\right),\\ f_{i}\left(X\right)\leq f_{i}\left(Y\right)\wedge \;\exists j\in \left(1,2,\ldots ,k\right),\\ f_{j}\left(X\right)<f_{j}\left(Y\right). \end{array}$$

**Definition 2 .**

(Pareto optimality). An obtained solution *X* is Pareto optimal iff

(3) $$\begin{array}{c} ∄Y\in R^{n}:Y≺X. \end{array}$$

**Definition 3 .**

(Pareto optimal set). The set of Pareto optimal solutions is called the Pareto optimal set (PS) iff it is defined as follows:

(4) $$\begin{array}{c} \text{PS}=\left\{X\in R^{n}|∄Y\in R^{n}:Y≺X\right\}. \end{array}$$

**Definition 4 .**

(Pareto optimal front). Given a Pareto optimal set (PS), the Pareto optimal front is defined as follows:

(5) $$\begin{array}{c} \text{PF}=\left\{F\left(X\right)X\in \text{PS}\right\}. \end{array}$$

### 2.2. Grid Mechanism

Grid mechanism [23, 26, 29] is introduced into DMOEOA due to its conciseness and high efficiency. In this mechanism, each individual is assigned a grid location in each dimension of the objective space. The grid mechanism is able to reflect the diversity and convergence of the obtained solutions. Some definitions of grid mechanism used in this work are as follows [26]:

**Definition 5 .**

(Grid boundary). min*f*_*i*_(*x*) and max*f*_*i*_(*x*) represent the minimum and maximum values of the *i*^th^ objective, respectively; the lower limit *l*_*i*_ and the upper limit *u*_*i*_ of the grid in the *i*^th^ objective space are as follows:

(6) $$\begin{array}{c} l_{i}=\min f_{i}\left(x\right)-\frac{\max f_{i}\left(x\right)-\min f_{i}\left(x\right)}{2\cdot \text{div}}, \end{array}$$

(7) $$\begin{array}{c} u_{i}=\max f_{i}\left(x\right)+\frac{\max f_{i}\left(x\right)-\min f_{i}\left(x\right)}{2\cdot \text{div}}, \end{array}$$

where div represents the number of divisions (i.e., grids) in the objective space in each dimension.

**Figure 1:**
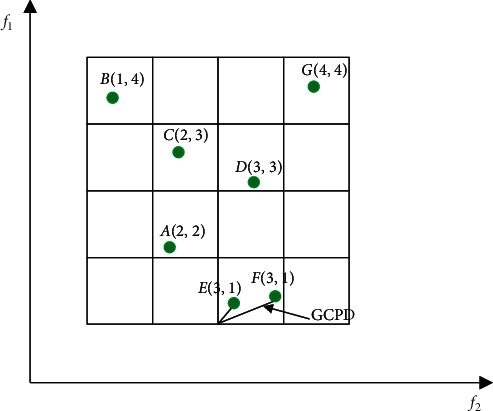
Illustration of grid mechanism in a biobjective space.

**Definition 6 .**

(Grid location). The grid location of an individual can be determined as follows:

(8) $$\begin{array}{c} {\text{GL}}_{i}\left(x\right)=\left[\frac{f_{i}\left(x\right)-l_{i}}{d_{i}}\right], \end{array}$$

where *d*_*i*_ is the width of the grid in the *i*^th^ objective, [·] represents the function of rounding up. For example, in Figure 1, the grid locations of individuals *B* and *C* are (1,  4) and (2,  3), respectively.

**Definition 7 .**

(Grid ranking). The grid ranking (GR) of an individual is defined as the summation of its grid location in each objective as follows:

(9) $$\begin{array}{c} \text{GR}\left(x\right)=\sum_{i=1}^{k}{\text{GL}}_{i}\left(x\right). \end{array}$$

The smaller the GR(*x*) value, the more individuals in the obtained solutions are dominated by an individual *x*. As shown in Figure 1, the grid ranking of *A* is 4; in contrast, the grid ranking of *D* is 6, which means that the individual *A* is closer to the true Pareto front than the individual *D*.

**Definition 8 .**

(Grid coordinate point distance). The normalized Euclidean distance between an individual and the minimal boundary point in its grid is called grid coordinate point distance (GCPD) , which is defined as follows:

(10) $$\begin{array}{c} \text{GCPD}\left(x\right)=\sqrt{\sum_{i=1}^{k}{\left[\frac{\left(f_{i}\left(x\right)-\left(l_{i}+\left({\text{GL}}_{i}\left(x\right)-1\right)\cdot d_{i}\right)\right)}{d_{i}}\right]}^{2}}. \end{array}$$

As for individuals who have the same grid ranking, the one who has a smaller GCPD value should be selected first. For example, in Figure 1, individuals *E* and *F* have the same GR value. However, the GCPD of the individual *E* is smaller than the individual *F*, so the individual *E* should be preferred. The general framework of the grid mechanism is shown in Algorithm 1.

## 3. The Proposed Algorithm

### 3.1. Equilibrium Optimizer (EO)

The equilibrium optimization algorithm is first proposed by Faramarzi et al. [12]. The equilibrium optimizer is inspired by the control volume mass balance model, which is applied to the estimation of dynamic and equilibrium states. In equilibrium optimizer, each individual (solution) with its concentration *C* (position) is regarded as a search agent. In EO, each individual in the population is similar to a solution and the individual's concentration is similar to a particle's position in the particle swarm optimization algorithm [1]. More information about EO may refer to [12]. Due to the simple principle, easy implementation, and fast convergence, EO has been widely applied to solve various single objective optimization problems, including economic dispatch [31], structural design optimization [32], and image segmentation [33]. The position updating formulation of EO is as follows [12]:

(11) $$\begin{array}{c} C=C_{e}+\left(C-C_{e}\right).F+\frac{G}{\lambda V}\left(1-F\right), \end{array}$$

where *V* is defined as unit, *C*_*e*_ refers to the equilibrium candidate, *F* and *G* represent exponential term and generation rate, respectively. *λ*=(*λ*_1_, *λ*_2_,…,*λ*_*n*_)^*T*^ is a random vector in the interval of [0, 1], *n* is the number of dimensions of the individual's concentration *C*.

#### 3.1.1. Equilibrium Pool *C*_*e*,pool_ and Equilibrium Candidate *C*_*e*_

The equilibrium state indicates the final convergence state of EO. At the beginning of the search process, there is no knowledge about the final equilibrium state, and the equilibrium candidate *C*_*e*_ is used to provide a search guide for individuals in the population. In equilibrium optimizer, equilibrium candidates are defined by the four best individuals selected according to their fitness value during the whole optimization process and an individual whose concentration is the average of the above four best individuals. The equilibrium pool consists of five individuals.

(12) $$\begin{array}{c} C_{e,\text{pool}}=\left\{C_{e\left(1\right)},C_{e\left(2\right)},C_{e\left(3\right)},C_{e\left(4\right)},C_{e\left(\text{ave}\right)}\right\}. \end{array}$$

However, as for multiobjective optimization problems, there is usually a set of alternative trade-offs between these objectives. We cannot sort the solutions based on their fitness value. Therefore, in DMOEOA, the classical external repository Rep in MOPSO [23] is used to construct the equilibrium pool. Solutions in the external repository are regarded as equilibrium candidates. The equilibrium pool in DMOEOA is shown below:

(13) $$\begin{array}{c} C_{e,\text{pool}}=\left\{\text{Rep}\right\}, \end{array}$$

where Rep represents the external repository, and the Rep is used to keep a historical record of the nondominated solutions found along the whole search process. Each individual in each iteration updates its concentration *C* (position) with roulette wheel selection among equilibrium candidates *C*_*e*_. The more equilibrium candidates with the same GR value in the equilibrium pool, the less likely they are to be selected to guide the particles in the population. The above selection method is able to maintain the diversity of the obtained solutions in the search process.

#### 3.1.2. Exponential Term *F*

The concentration updating rule is mainly controlled by the exponential term *F*.

(14) $$\begin{array}{c} F=e^{-\lambda \left(t-t_{0}\right)}, \end{array}$$

(15) $$\begin{array}{c} t={\left(1-\frac{\text{iter}}{\text{IT}}\right)}^{\left(a_{2}\left(\text{iter}/\text{IT}\right)\right)}, \end{array}$$

where *t* is the function of iterations, *t* decreases with the number of iterations, iter and IT represent the current iteration and the maximum iteration, respectively. *a*_2_ is a constant value which controls the exploitation ability of EO. With the aim of achieving high convergence by slowing down the search speed, *t*_0_ is defined as follows:

(16) $$\begin{array}{c} t_{0}=\frac{1}{\lambda }\ln\left(-a_{1}\text{sign}\left(r_{0}-0.5\right)\left[1-e^{-\lambda t}\right]\right)+t, \end{array}$$

where *a*_1_ is a constant value that affects the exploration ability, sign(*r*_0_ − 0.5) is applied to control the direction of exploration and exploitation, *r*_0_ is a random number in [0, 1]. In this work, the values of *a*_1_ and *a*_2_ are set to 2 and 1, respectively. The selection of the two values is consistent with the original EO algorithm. Therefore, the exponential term *F* can be formulated as follows:

(17) $$\begin{array}{c} F=a_{1}\text{sign}\left(r_{0}-0.5\right)\left(e^{-\lambda t}-1\right). \end{array}$$

#### 3.1.3. Generation Rate *G*

Generation rate plays an important role in the equilibrium algorithm. It is used to improve the exploitation ability of EO.

(18) $$\begin{array}{c} G=G_{0}e^{-\kappa \left(t-t_{0}\right)},\\ G_{0}=\text{GCP}\left(C_{e}-\lambda C\right),\\ \text{GCP}=\left\{\begin{array}{cc} 0.5r_{1}, & r_{2}\geq \text{GP},\\ 0, & r_{2}<\text{GP}, \end{array}\right. \end{array}$$

where *G*_0_ represents the initial value. GCP is called the generation rate control probability. GP represents the generation probability, which is set to 0.5 according to the original EO algorithm. *r*_1_ and *r*_2_ are two random numbers in [0, 1]. *κ* indicates the decay vector. This study assumes *κ*=*λ*. Thus, the generation rate can be formulated as follows:

(19) $$\begin{array}{c} G=G_{0}F. \end{array}$$

### 3.2. Layered Disruption Method (LDM)

Inspired by the disruption phenomenon of astrophysics, a novel operator named “Disruption” and its variants are introduced into single objective evolutionary algorithms [11, 34, 35]. In this paper, a layered disruption method is integrated into a multiobjective equilibrium optimization algorithm to enhance its exploration and exploitation abilities.

#### 3.2.1. Disruption Phenomenon

**Figure 2:**
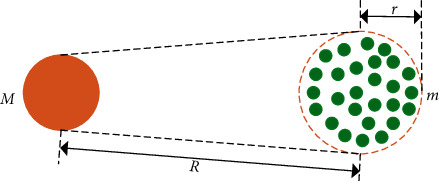
Illustration of disruption phenomenon.

“When a swarm of gravitationally bound particles having a total mass, *m*, approaches too close to a massive object, *M*, the swarm tends to be torn apart. The same thing can happen to a solid body held together by gravitational forces when it approaches a much more massive object” [36]. This is called disruption phenomena. The disruption phenomenon is originated from astrophysics [36]. As shown in Figure 2, the swarm will be torn apart when the following condition is satisfied [11]:

(20) $$\begin{array}{c} \frac{R^{3}}{r^{3}}<\frac{2M}{m}, \end{array}$$

where *R* is the distance between the center of mass of the swarm *m* and the mass *M*, and *r* represents the radius of the swarm *m*.

#### 3.2.2. Layered Disruption Condition

In order to simulate the disruption phenomenon, individuals in the population Pop with the same GR value are treated as one group, and different groups have different disruption conditions. It is different from Liu et al. [11], Sarafrazi et al. [34], and Ding et al. [35]. All individuals are treated as one group. Here, we define the disruption coefficient *Q*_*i*_ as follows:

(21) $$\begin{array}{c} Q_{i}=\exp\left[-{\left(\frac{i}{\gamma }\right)}^{2}\right], \end{array}$$

where *γ* is the number of groups. *i* represents the *i*^th^ index after sorting all groups by increasing order according to GR values.

In the *i*^th^ group, individuals with the *S*_*i*_ smallest GCPD values are treated as a whole and denoted as the mass *M*. Other individuals who will be disrupted have the total mass *m*. *S*_*i*_ is defined as follows:

(22) $$\begin{array}{c} S_{i}=\left[Q_{i}\cdot U_{i}\right], \end{array}$$

where *U*_*i*_ is the number of individuals in the *i*^th^ group, and [·] is the function of rounding up.

#### 3.2.3. Disruption Operator

When the individual satisfies the disruption condition, a random number which obeys the Cauchy distribution is utilized to disrupt the individual. The cauchyrnd is defined as follows:

(23) $$\begin{array}{c} f\left(x\right)=\frac{1}{\pi \cdot \left(1+x^{2}\right)},\;x\in \left[-\infty ,+\infty \right]. \end{array}$$

The disruption equation is as follows:

(24) $$\begin{array}{c} C_{j}\left(\text{iter}\right)=\left(\frac{\text{iter}}{\text{IT}}\right)\cdot C_{j}\left(\text{iter}\right)+\text{Cau}\cdot \left(1-\frac{\text{iter}}{\text{IT}}\right)\cdot C_{j}\left(\text{iter}\right), \end{array}$$

where *C*_*j*_ is the position vector of individual *j*, iter is the current iteration, IT represents the max iteration. Cau refers to the disruption operator which is a matrix consisting of a set of Cauchy random numbers. It is worth noting that different dimensions of individual *j* have different Cauchy random numbers, which is different from Liu et al. [11]. All dimensions of individual *j* have the same cauchyrnd.

We can observe that the individual with large GR value and GCPD value is more likely to be disrupted to explore in a wide region at the early stage. As the number of iterations increases, the individual will fully exploit its surrounding area. Therefore, the disruption method proposed in this paper is able to enhance the exploration and exploitation abilities of the proposed algorithm. The general framework of the layered disruption method is shown in Algorithm 2.

### 3.3. The Pseudocode of the DMOEOA Algorithm

**Figure 3:**
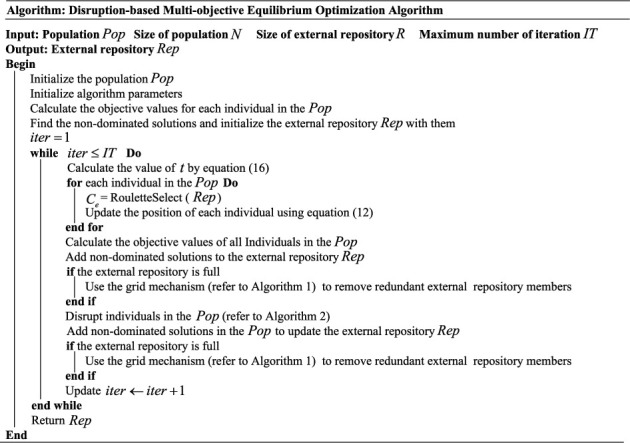
Pseudocode of the DMOEOA algorithm.

The pseudocode of the DMOEOA algorithm is shown in Figure 3.

### 3.4. Computational Complexity Analysis of the DMOEOA Algorithm

The computational complexity of an algorithm indicates the number of resources required to run it; the computational complexity of an algorithm can reflect the performance of the algorithm. *N* refers to the number of individuals in the population and *K* represents the number of objectives. The computational complexity of the main steps of DMOEOA is shown in Table 1.

Therefore, the computational complexity of DMOEOA is of *O*(KN^2^). The computational complexity of DMOEOA is the same as the algorithms employed to compare with DMOEOA in this paper, including MOPSO, MOALO, NSGAII, MOWOA, and MOGWO.

## 4. Simulation Results and Discussion

### 4.1. Parameter Setting and Instances

In this section, three kinds of standard benchmark test suites including ZDT suites [37], DTLZ suites [38], and UF suites [39] are utilized to validate the performance of the proposed DMOEOA algorithm. The optimal Pareto fronts of these test functions include continuous, discontinuous, convex, and concave. Five multiobjective optimization algorithms, including MOPSO, MOALO, MOWOA, NSGAII, and MOGWO, are employed to compare with DMOEOA. The parameters of algorithms shown in Table 2 are chosen. These parameters are selected in accordance with the original algorithms. For all of the following simulation experiments, the maximum number of iterations and populations is set to 300 and 200, respectively. As for ZDT suites [37] and UF suites [39], the dimension of the search space is set to 30 and the dimension of the search space of DTLZ suites [38] is set to 12. To eliminate the randomness of the results, each algorithm runs 30 times on each benchmark test function.

### 4.2. Performance Metrics

In order to minimize the distance of the Pareto front produced by DMOEOA with respect to the optimal Pareto front and maximize the diversity of solutions found, two performance metrics are employed to quantify the performance of multiobjective optimization algorithms, including Inverted Generational Distance (IGD) [40] and metric of Delta (Δ) [19].

The performance metrics of Inverted Generational Distance and Delta are formulated as follows:

(25) $$\begin{array}{c} \text{IGD}=\frac{1}{P}\cdot \sqrt{\sum_{i=1}^{P}D_{i}^{2}}, \end{array}$$

where *P* represents the number of true Pareto optimal solutions, *D*_*i*_ indicates the Euclidean distance between the *i*^th^ true Pareto optimal solution and its nearest solutions in the external repository. In addition to reflecting the convergence of the obtained solutions, IGD can reflect the uniformity and coverage of the obtained solutions. The smaller the IGD value, the better coverage and convergence of the obtained solutions.

(26) $$\begin{array}{c} \Delta =\frac{d_{f}+d_{l}+\overset{E-1}{\underset{i=1}{\sum }}\left|d_{i}-\overset{¯}{d}\right|}{d_{f}+d_{l}+\left(E-1\right)\bar{d}}, \end{array}$$

where *d*_*i*_ is the Euclidean distance between consecutive solutions in the obtained solutions and $\bar{d}$ is the mean of these distances. *d*_*f*_ and *d*_*l*_ represent the extreme solutions and the boundary solutions of the obtained solutions, respectively. *E* is the number of obtained solutions. The smaller the Delta value, the better the diversity of the solution set.

### 4.3. Discussion and Analysis

**Figure 4:**
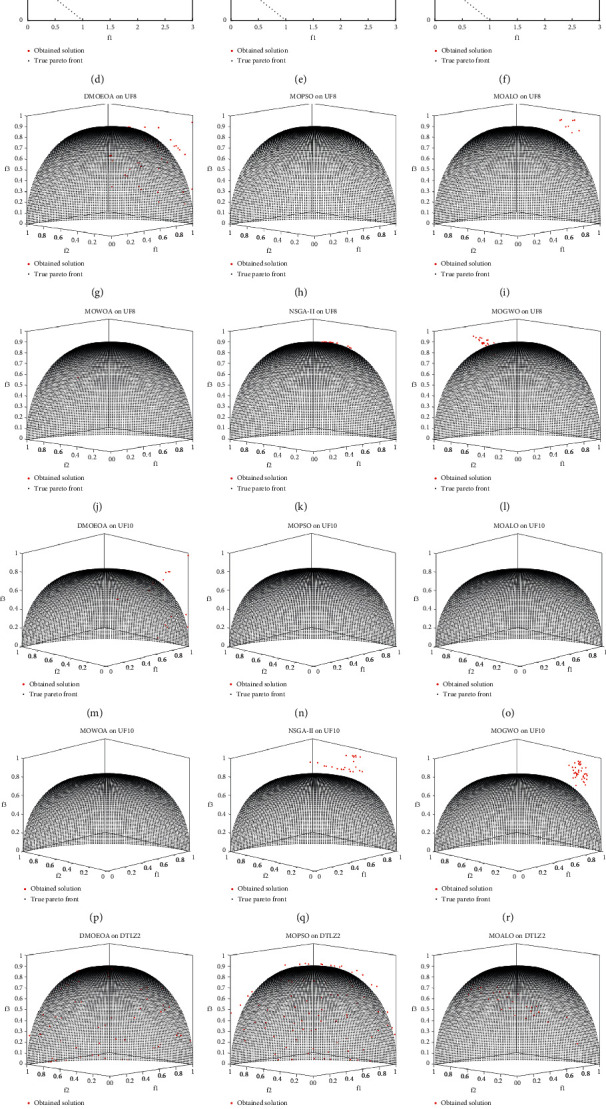
The shape of best Pareto optimal fronts obtained by DMOEOA, MOPSO, MOALO, MOWOA, NSGAII, and MOGWO on some hard test problems.

**Figure 5:**
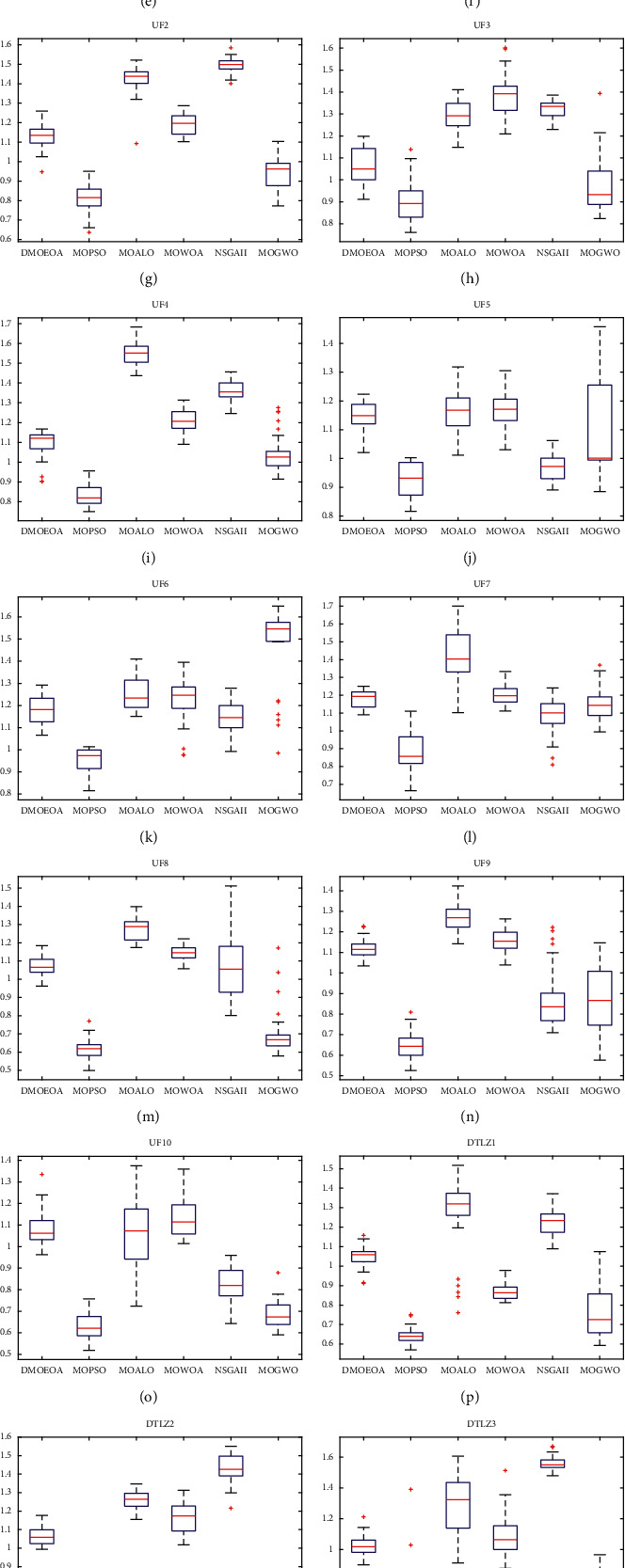
Boxplot of the statistical results for Delta obtained by DMOEOA, MOPSO, MOALO, MOWOA, NSGAII, and MOGWO.

This section provides the statistical results of DMOEOA and five multiobjective optimization algorithms, including MOPSO, MOALO, MOWOA, NSGAII, and MOGWO, for IGD metric and Delta metric. The results obtained by those six algorithms upon test functions are shown in Tables 3 and 4 and Figures 4 and 5. The best value is shown in bold. In addition, the Wilcoxon rank-sum test is employed to compare the IGD results obtained by DMOEOA, and those five compared algorithms at a significance level of 0.05. The IGD results for two-objective test functions and three-objective test functions are shown in Tables 3 and 4, respectively, in which the “+/=/−” represent the proposed algorithm is better than, similar to, or worse than its corresponding competitor, respectively. The results are represented by “*w*/*t*/*l*“, which means that compared to the competitor, DMOEOA wins on *w* test functions, ties on *t* test functions, and loses on *l* test functions.

The statistical results shown in Table 3 indicate that DMOEOA provides better performance in convergence and coverage than MOPSO, MOALO, MOWOA, and NSGA-II. From Figure 5, we can observe that MOPSO shows better diversity of obtained solutions than other five algorithms on ZDT1. The statistical results of the algorithms on ZDT2 and ZDT3 for IGD in Table 3 show that the proposed DMOEOA algorithm provides better results on average and standard deviation of IGD than the other five algorithms. IGD is a performance metric that reflects the convergence and coverage performance of an algorithm, which means that the DMOEOA algorithm provides better convergence and coverage of obtained solutions on ZDT3. In Figure 5, the boxplot of Delta on ZDT2 indicates that DMOEOA and MOALO show similar performance in a diversity of obtained solutions, and the boxplot of Delta on ZDT3 suggests that the DMOEOA algorithm has better performance in a diversity of obtained solutions than MOALO, MOWOA, and MOGWO.

As shown in Table 3, the statistical results of the six algorithms on ZDT4 and ZDT6 test problems for IGD show that the proposed DMOEOA algorithm is able to outperform the other five algorithms on average, and the Wilcoxon rank-sum test results indicate that the proposed algorithm has superiority in both coverage and convergence performance. From Figure 5, we can observe that although MOPSO and NSGAII outperform the other four algorithms in a diversity of obtained solutions on ZDT4 and ZDT6, the above two algorithms show poor convergence ability on ZDT4 and ZDT6 test functions.

The statistical results of the algorithms on UF1 and UF2 for IGD in Table 3 show that the proposed DMOEOA algorithm provides better results on average and standard deviation of IGD than the other five algorithms, which means that the DMOEOA algorithm shows better performance in convergence and coverage on UF1 and UF2. As shown in Figure 5, the boxplot of Delta on UF1 indicates that DMOEOA provides better performance in the diversity of obtained solutions than MOALO, NSGA-II, and MOGWO. In contrast, the boxplot of Delta on UF2 indicates that MOPSO shows better performance in diversity than the other five algorithms.

The best results on average and standard deviation of IGD for UF3 belong to MOGWO and MOWOA, respectively (see Table 3). The statistical results of the algorithms on UF4 for IGD (see Table 3) indicate that DMOEOA shows better performance in convergence and coverage than the other five algorithms. The boxplot of Delta on UF3 and UF4 shown in Figure 5 indicates that DMOEOA has better performance in diversity than MOALO, MOWOA and NSGA-II.

UF5 test function has discontinuous Pareto optimal front. As shown in Figure 4, the best obtained optimal Pareto fronts of the six algorithms for UF5 suggest that the nondominated solutions obtained by DMOEOA are more uniformly distributed than the other five algorithms. According to the Wilcoxon rank-sum test results, the convergence ability of the proposed algorithm on UF5 is similar to that of MOGWO. The statistical results of the algorithms for the IGD metric on UF6 (see Table 3) show that the convergence and coverage performance of the proposed algorithm DMOEOA is similar to that of MOPSO, MOALO, and MOWOA.

UF7 benchmark has a linear Pareto optimal front. Compared to test functions with disconnected Pareto optimal fronts, it is easier for algorithms to obtain well-distributed solutions on the UF7 test problem. The statistical results of the algorithm for the IGD metric (see Table 3) on UF7 prove that the DMOEOA algorithm has better performance in convergence and coverage than MOPSO, MOALO, and NSGAII. As depicted in Figure 5, the boxplot of Delta on UF7 suggests that the DMOEOA algorithm shows better performance in the diversity of obtained nondominated solutions than MOALO, MOGWO, and MOWOA.

UF8, UF9, and UF10 are triobjective test problems, and these three benchmarks have complex Pareto optimal fronts, which make them challenging for all the six algorithms. As shown in Figure 4, the best obtained Pareto optimal front of DMOEOA on UF8 is more distributed than the other five algorithms. Meanwhile, the statistical results shown in Table 4 suggest that DMOEOA provides better results on average and standard deviation of IGD. It can be stated that the proposed DMOEOA algorithm has better performance in both convergence and distribution than the other five algorithms on the UF8 test problem. From Figure 4, we can observe that all the six algorithms show poor convergence and distribution on UF9. Compared to the other five algorithms, the DMOEOA has superiority in both coverage and convergence of obtained solutions on UF10 according to the Wilcoxon rank-sum test results shown in Table 4.

DTLZ1 and DTLZ2 are triobjective test problems and they have multiple local Pareto optimal fronts. The statistical results of the algorithm for IGD on DTLZ1 in Table 4 show that the proposed DMOEOA algorithm performs better in convergence than the other five algorithms. As shown in Figure 4, the best obtained optimal Pareto front of NSGA-II on DTLZ2 is far from the true Pareto optimal front. In contrast, the obtained optimal Pareto fronts of DMOEOA and MOPSO are more uniformly distributed than the other four algorithms. Meanwhile, the statistical results of the DMOEOA for IGD on DTLZ2 in Table 4 prove that DMOEOA and MOPSO have superiority in convergence ability.

Both DTLZ3 and DTLZ4 have concave Pareto optimal fronts. The statistical results of the algorithms on DTLZ3 and DTLZ4 for IGD (see Table 4) show that DMOEOA shows better results on average and standard deviation of IGD than the other five algorithms. MOPSO and MOGWO provide better performance in the diversity of obtained solutions than the other four algorithms on both DTLZ3 and DTLZ4 (see Figure 5).

DTLZ5 and DTLZ6 are both three-objective test problems with degenerate Pareto optimal fronts. As shown in Table 4, the statistical results of the algorithms for IGD on DTLZ5 indicate that DMOEOA has a similar performance in convergence and coverage with MOPSO and MOGWO. MOPSO shows better performance in both diversity and convergence of obtained solutions than DMOEOA, MOPSO, MOALO, and NSGA-II on DTLZ6 (see Table 4 and Figure 5).

DTLZ7 is disconnected in both the Pareto optimal set and the Pareto optimal front. In Table 4, the statistical results of the algorithms for IGD on DTLZ7 suggest that DMOEOA provides better results on average and standard deviation of IGD than the other five algorithms, which means that the DMOEOA algorithm shows superiority in both convergence and coverage ability on DTLZ7, and the boxplot of Delta on DTLZ7 indicates that DMOEOA shows better performance in the diversity of obtained solutions than MOALO, MOWOA, and NSGA-II (see Figure 5).

The above results demonstrate that the DMOEOA algorithm is able to show competitive and promising results on multiobjective test functions, especially for three-objective test problems, and the test results indicate that DMOEOA does exhibit better performances in these problems with a better balance between convergence and distribution. The statistical results for IGD demonstrate the high convergence ability of DMOEOA. The layered disruption method plays an important role in improving the convergence and distribution performance of DMOEOA. LDM can prompt the population to conduct extensive searches in each iteration. As the number of iterations increases, the individual will fully exploit its surrounding area. Thus, the exploration and exploitation ability of the proposed algorithm can be enhanced.

### 4.4. Analysis of Layered Disruption Method (LDM)

**Figure 6:**
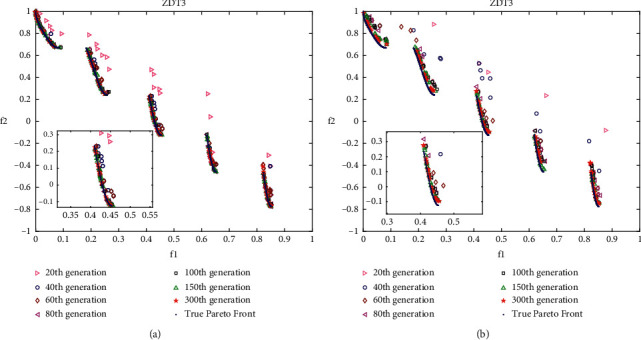
Pareto solutions of different intermediate generations on ZDT3 obtained by DMOEOA (a) and MOEOA (b).

**Figure 7:**
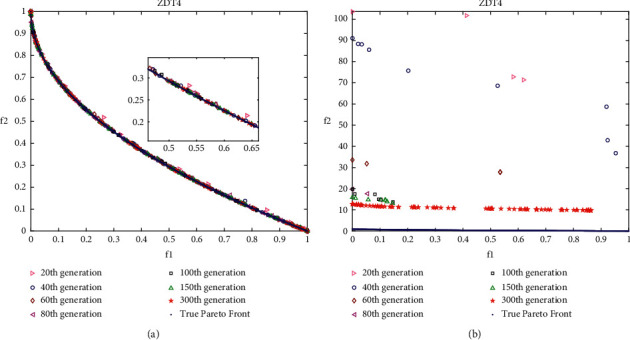
Pareto solutions of different intermediate generations on ZDT4 obtained by DMOEOA (a) and MOEOA (b).

**Figure 8:**
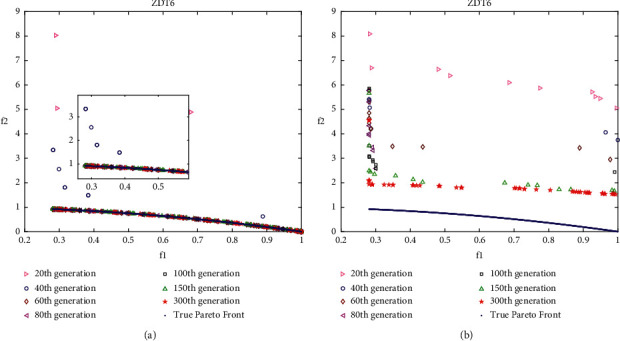
Pareto solutions of different intermediate generations on ZDT6 obtained by DMOEOA (a) and MOEOA (b).

**Figure 9:**
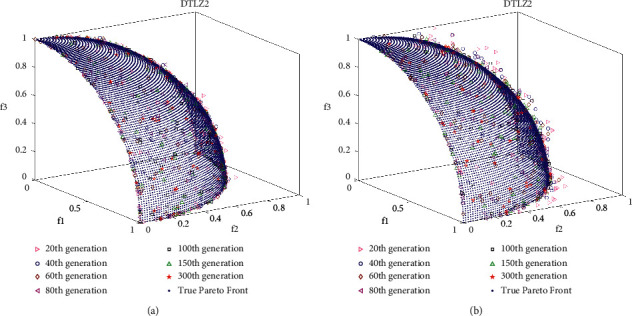
Pareto solutions of different intermediate generations on DTLZ2 obtained by DMOEOA (a) and MOEOA (b).

In this work, the layered disruption method (LDM) is introduced into DMOEOA with the aim of enhancing its exploration and exploitation abilities. Thus, it is important to investigate the impact of the LDM on DMOEOA. In this section, ZDT3, ZDT4, ZDT6, and DTLZ2 test problems which include discontinuous, convex, and concave Pareto optimal fronts are employed as test instances. Different intermediate generations of nondominated solutions obtained by DMOEOA and DMOEOA without LDM (denoted as MOEOA) are recorded to see the impact of LDM on the proposed algorithm. The numbers of recorded intermediate generations include 20, 40, 60, 80, 100, 150, 300. Other parameters of the DMOEOA algorithm are the same as in Table 1. Simulation results are depicted in Figures 6–9. The results of the algorithms on ZDT3 depicted in Figure 6 show that DMOEOA is able to find the true optimal Pareto front after the 60th generation. In contrast, MOEOA cannot completely converge to the true optimal solutions even in the 300th generation. As shown in Figure 7, the DMOEOA converges to the true Pareto front after the 80th generation on ZDT4. By comparison, MOEOA shows poor convergence and distribution ability on ZDT4. Similarly, simulation results of the algorithms on ZDT6 in Figure 8 indicate that DMOEOA converges to the optimal Pareto front after the 60th generation. As for MOEOA, there are still some poor solutions in the 300th generation. In addition, as shown in Figure 8, the distribution of best obtained optimal Pareto solutions of DMOEOA is better than MOEOA. Compared to MOEOA, the DMOEOA is able to find the optimal solutions of DTLZ2 shown in Figure 9 faster. From simulation results depicted in Figures 6–9, we can observe that the LDM is able to enhance the exploration and exploitation ability of the proposed algorithm.

### 4.5. Parametric Study

The proposed DMOEOA algorithm introduces the grid mechanism. Meanwhile, the layered disruption method is based on the grid mechanism, and the number of grid divisions is a key parameter in the grid mechanism. Therefore, it is necessary to investigate the effect of grid division in the performance of the DMOEOA algorithm. In this section, UF {5, 6, 7, 8, 9, 10} test suites are utilized as test instances. Inverted Generational Distance (IGD) is employed as the performance metric. We performed runs using different numbers of grid division to see the effect of this parameter in the performance of the DMOEOA algorithm. The number of grid divisions ranges from 5 to 15. Other parameters of the DMOEOA algorithm are the same as in Table 1. To eliminate the randomness of the results, for each given grid division, the proposed algorithm runs 30 times on each benchmark test function.

**Figure 10:**
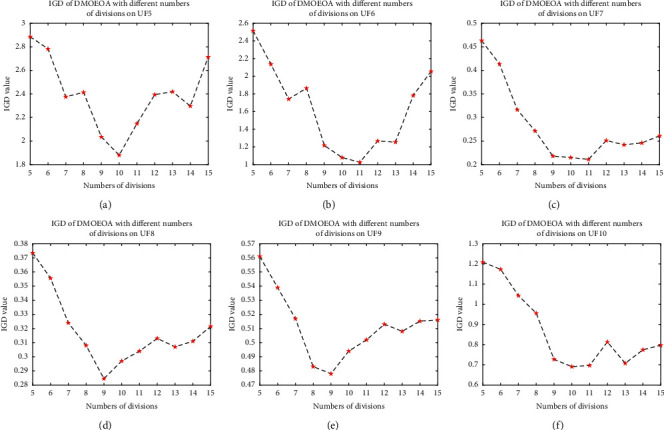
IGD of DMOEOA with different numbers of division on UF {5, 6, 7, 8, 9, 10} test suites. (a) IGD of DMOEOA with different numbers of divisions on UF5. (b) IGD of DMOEOA with different numbers of divisions on UF6. (c) IGD of DMOEOA with different numbers of divisions on UF7. (d) IGD of DMOEOA with different numbers of divisions on UF8. (e) IGD of DMOEOA with different numbers of divisions on UF9. (f) IGD of DMOEOA with different numbers of divisions on UF10.

As shown in Figure 10, with the increase of grid divisions from 5 to 9, mean IGD values of DMOEOA on UF {5, 6, 8, 9} test instances decreased gradually, then the mean values of IGD increased with the number of divisions. As for UF7 and UF10 test instances, the mean IGD values increased slowly with the numbers of grid divisions from 9 to 15. From Figure 10, we can observe that too many or too few divisions will affect the performance of the algorithm. The appropriate number of grid divisions is beneficial to improve the convergence and coverage ability of the proposed algorithm. In general, DMOEOA performs well at div ∈ [9,11] for both biobjective and triobjective test problems.

## 5. Application in Structural Optimization of an Elastic Truss

**Figure 11:**
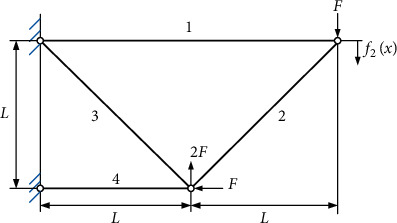
Structure of the 4-bar truss.

In this section, the proposed DMOEOA algorithm is applied to the structural optimization of a 4-bar elastic truss as a demonstration. The 4-bar elastic truss design optimization problem is a well-known engineering problem in the structural optimization field [41]. The structure of the 4-bar truss is shown in Figure 11.

The truss is designed with joint displacement and structural volume as the objectives. And areas of member cross sections are set as design variables. Mathematically, this engineering problem is as follows:

(27) $$\begin{array}{c} \min f_{1}\left(x\right)=L\left(2x_{1}+\sqrt{2}x_{2}+\sqrt{x_{3}}+x_{4}\right),\\ \min f_{2}\left(x\right)=\frac{\text{FL}}{E}\left(\frac{2}{x_{1}}+\frac{2\sqrt{2}}{x_{2}}-\frac{2\sqrt{2}}{x_{3}}+\frac{2}{x_{4}}\right),\\ \text{S}.\text{t}.\;\left\{\begin{array}{c} \frac{F}{\sigma }\leq x_{1}\leq \frac{3F}{\sigma },\\ \frac{\sqrt{2}F}{\sigma }\leq x_{2}\leq \frac{3F}{\sigma },\\ \frac{\sqrt{2}F}{\sigma }\leq x_{3}\leq \frac{3F}{\sigma },\\ \frac{F}{\sigma }\leq x_{4}\leq \frac{3F}{\sigma }, \end{array}\right. \end{array}$$

where *F*=10 kN, *E*=2 · 10^5^ kN/cm^2^, *L*=200 cm,*σ*=10 kN/cm^2^. *f*_1_(*x*) and *f*_2_(*x*) represent the structural volume and joint displacement of the truss, respectively. *x*_*i*_ (*i*=1,2,3,4) is the area of cross section of *i*^th^ member.

**Figure 12:**
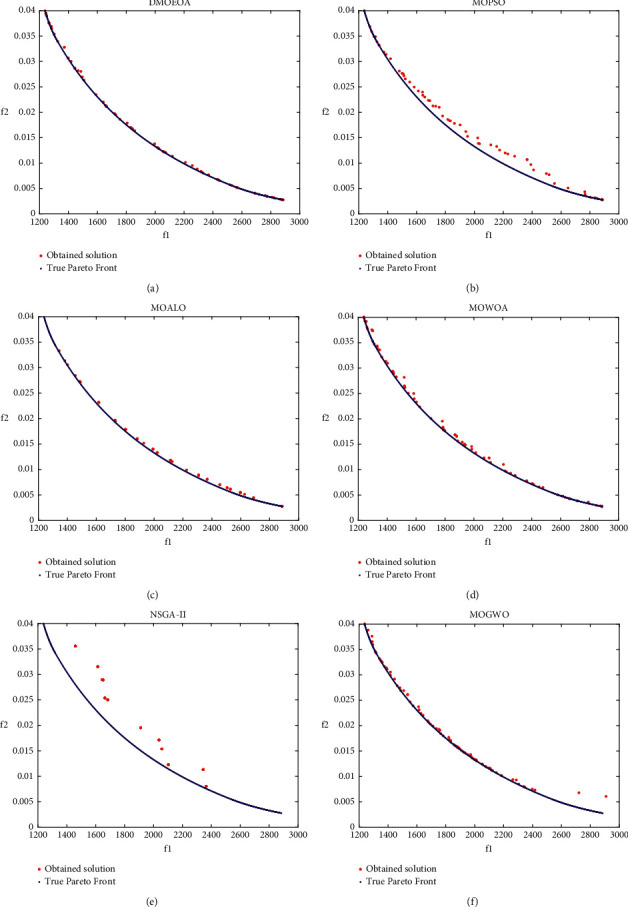
The shape of best Pareto optimal fronts obtained by (a) DMOEOA, (b) MOPSO, (c) MOALO, (d) MOWOA, (e) NSGAII, and (f) MOGWO on the structural optimization problem.

IGD metric is utilized as the performance metric. The above five algorithms including MOPSO, MOALO, MOWOA, NSGAII, and MOGWO are employed to be compared with DMOEOA. The maximum number of iterations and populations is set to 100 and 100, respectively. To eliminate the randomness of the results, each algorithm runs 30 times. The statistical results obtained by those six algorithms upon the above structural optimization problem are shown in Table 5 and Figure 12.

The statistical results shown in Table 5 suggest that DMOEOA provides better results on best, average, and standard deviation of IGD than the other five algorithms. Although the better result on the worst of IGD is obtained by MOPSO, the superiority of DMOEOA in convergence is significant. From Figure 12, we can observe that DMOEOA is able to converge to the true optimal Pareto front. In contrast, NSGAII and MOALO show the poor distribution of obtained solutions on this structural optimization problem.

## 6. Conclusion

This paper proposes a disruption-based multiobjective equilibrium optimization algorithm (DMOEOA). This algorithm integrates a layered disruption method proposed in this work. Layered disruption method (LDM) is proposed to enhance the exploration and exploitation abilities of the proposed algorithm. To validate the effectiveness of the DMOEOA algorithm, three kinds of benchmark test suites have been selected with five different multiobjective optimization algorithms which include well-known algorithms and state-of-the-art algorithms. The test results suggest that the DMOEOA algorithm is able to show well performance in these test problems with a better balance between convergence and distribution. The impact of the layered disruption method is analyzed. In addition, we discuss the influence of division numbers on the performance of the proposed DMOEOA algorithm. Moreover, the new proposed algorithm is also applied for solving the structural optimization problem of a four-bar elastic truss. Compared with the other five optimizers, the results show that DMOEOA is not only an algorithm with well performance for benchmark test functions but also expected to have a wide application in engineering design optimization problems. Future research should focus on applying the proposed DMOEOA algorithm to handle constrained real engineering problems and many-objective optimization problems (Table 6).

## Figures and Tables

**Algorithm 1:**
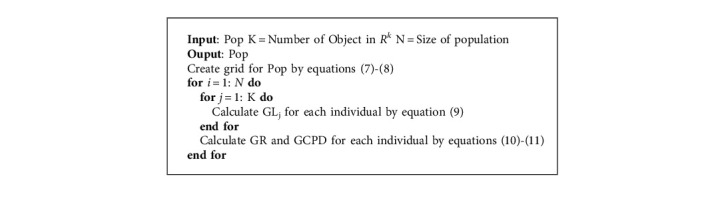
Grid Mechanism.

**Algorithm 2:**
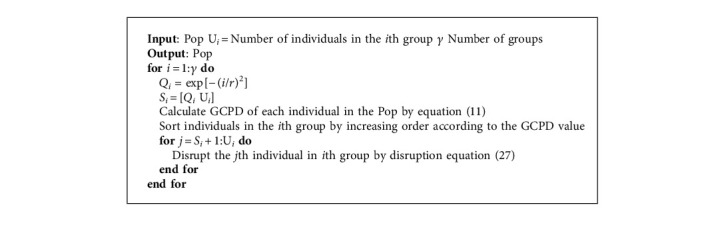
Layered Disruption Method.

**Table 1:** Computational complexity about DMOEOA.

Step	Computational complexity
Position updating	*O*(KN)
Grid mechanism	*O*(KN^2^)
Layered disruption method	*O*(KN^2^)
Maintenance of external repository	*O*(KN^2^)

**Table 2:** Parameters of five multiobjective optimization algorithms.

Algorithm	Parameter	Value
DMOEOA	Division	10
	a1	2
	a2	1
	GP	0.5

MOPSO	nGrid	10
	Inertia weight	0.5
	c1	1
	c2	2

MOWOA	*h*	10

NSGA-II	Crossover probability	1
	Mutation probability	0.1

MOGWO	Alpha	0.1
	Beta	4
	Gamma	2
	nGrid	10

**Table 3:** IGD metric results obtained by DMOEOA and five multiobjective algorithms on two-objective test functions.

Problem	Metric	DMOEOA	MOPSO	MOALO	NSGAII	MOWOA	MOGWO
ZDT1	Ave	1.30 *E*−02	1.86 *E*−02	1.91 *E*−01	2.12 *E* + 00	1.62 *E−*02	**9.46* *E−03**
	Std	**1.22* E−*03**	5.27 *E*−03	7.20 *E*−02	2.43 *E*−01	8.63 *E*−03	2.12 *E*−03
			+	+	+	+	−

ZDT2	Ave	**9.98* E*−03**	4.30 *E*−02	4.93 *E*−01	3.31 *E* + 00	1.15 *E*−02	1.04 *E*−02
	Std	**1.01* E*−03**	1.18 *E*−01	1.48 *E*−01	2.61 *E*−01	1.56 *E*−03	3.37 *E*−03
			+	+	+	+	+

ZDT3	Ave	**1.47* E*−02**	5.84 *E*−02	1.22 *E*−01	2.84 *E*−01	1.72 *E*−02	1.68 *E*−02
	Std	**2.18* E*−03**	4.79 *E*−02	2.81 *E*−02	5.90 *E*−02	3.08 *E*−03	5.98 *E*−03
			+	+	+	+	=

ZDT4	Ave	**2.21* E*−02**	5.78 *E* + 00	2.90 *E* + 01	9.69 *E* + 00	2.29 *E*−02	1.02 *E* + 01
	Std	2.01 *E*−02	1.92 *E* + 00	1.12 *E* + 01	3.49 *E*−01	**7.75* E*−03**	1.55 *E* + 01
			+	+	+	+	+

ZDT6	Ave	**2.68* E*−02**	2.77 *E* + 00	5.82 *E*−01	6.17 *E* + 00	1.75 *E*−01	1.99 *E*−01
	Std	5.31 *E*−02	1.99 *E* + 00	7.81 *E*−02	2.38 *E* + 00	**3.80* E*−03**	7.99 *E*−02
			+	+	+	+	+

UF1	Ave	**1.13* E*−01**	2.86 *E*−01	2.04 *E*−01	1.25 *E* + 00	1.69 *E*−01	1.34 *E*−01
	Std	**1.95* E*−02**	1.06 *E*−01	3.15 *E*−02	1.28 *E*−01	2.50 *E*−02	4.53 *E*−02
			+	+	+	+	+

UF2	Ave	**5.31* E*−02**	2.00 *E*−01	2.12 *E*−01	4.99 *E*−01	9.60 *E*−02	7.45 *E*−02
	Std	**6.98* E*−03**	3.89 *E*−02	3.33 *E*−02	5.41 *E*−02	7.79 *E*−03	8.91 *E*−03
			+	+	+	+	+

UF3	Ave	5.81 *E*−01	6.51 *E*−01	6.80 *E*−01	1.26 *E* + 00	3.08 *E*−01	**2.77* E*−01**
	Std	4.90 *E*−02	1.68 *E*−01	1.74 *E*−01	1.07 *E*−01	**1.30* E*−02**	3.75 *E*−02
			+	+	+	−	−

UF4	Ave	**6.48* E*−02**	9.79 *E*−02	9.12 *E*−02	1.89 *E*−01	6.92 *E*−02	6.80 *E*−02
	Std	**4.60* E*−03**	6.68 *E*−03	1.77 *E*−02	5.76 *E*−03	5.92 *E*−03	5.28 *E*−03
			+	+	+	+	+

UF5	Ave	1.88 *E* + 00	2.44 *E* + 00	2.16 *E* + 00	**1.23* E*** **+** **00**	2.23 *E* + 00	1.93 *E* + 00
	Std	5.08 *E*−01	8.97 *E*−01	1.98 *E*−01	**1.46* E*−01**	3.56 *E*−01	6.38 *E*−01
			+	+	−	+	=

UF6	Ave	1.08 *E* + 00	1.12 *E* *+* 00	1.10 *E* + 00	5.63 *E*−01	1.14 *E* + 00	**3.48* E*−01**
	Std	2.76 *E*−01	2.62 *E*−01	3.02 *E*−01	6.80 *E*−02	5.06 *E*−01	**1.48* E*−02**
			=	=	−	=	−

UF7	Ave	2.18 *E*−01	2.87 *E*−01	2.73 *E*−01	2.83 *E*−01	2.68 *E*−01	**8.20* E*−02**
	Std	8.66 *E*−02	1.34 *E*−01	9.77 *E*−02	1.33 *E*−01	1.25 *E*−01	**1.13* E*−02**
			+	+	+	=	−
	*w*\*t*\*l*		11\1\0	11\1\0	10\0\2	9\2\1	6\2\4

**Table 4:** IGD metric results obtained by DMOEOA and five multiobjective algorithms on three-objective test functions.

Problem	Metric	DMOEOA	MOPSO	MOALO	NSGAII	MOWOA	MOGWO
UF8	Ave	**2.97* E*−01**	1.31 *E+*00	7.24 *E*−01	4.37 *E*−01	6.38 *E*−01	6.75 *E*−01
	Std	**2.97* E*−02**	2.88 *E−*01	1.60 *E*−01	4.92 *E*−02	1.17 *E*−01	2.73 *E*−01
			+	+	+	+	+

UF9	Ave	4.78 *E*−01	1.50 *E* + 00	6.64 *E*−01	3.66 *E*−01	8.15 *E*−01	**2.69* E*−01**
	Std	5.53 *E*−02	2.57 *E*−01	1.26 *E*−01	**2.22* E*−02**	9.63 *E*−02	1.14 *E*−01
			+	+	−	+	−

UF10	Ave	**6.91* E*−01**	7.69 *E* + 00	4.44 *E* + 00	7.09 *E*−01	5.33 *E* + 00	2.71 *E* + 00
	Std	**7.46* E*−02**	1.32 *E* + 00	9.25 *E*−01	8.04 *E*−02	6.13 *E*−01	9.23 *E*−01
			+	+	+	+	+

DTLZ1	Ave	**5.45* E*** **+** **01**	8.82 *E* + 01	5.48 *E* + 01	3.95 *E* + 02	8.98 *E* + 01	7.77 *E* + 01
	Std	5.34 *E* + 00	6.40 *E* + 00	3.93 *E* + 01	3.72 *E* + 01	**3.06* E*** **+** **00**	3.26 *E* + 01
			+	=	+	+	+

DTLZ2	Ave	9.16 *E*−02	**7.84* E*−02**	2.51 *E*−01	3.34 *E* + 00	8.89 *E*−02	4.06 *E−*01
	Std	5.02 *E*−03	**4.33* E*−03**	5.51 *E*−02	3.43 *E*−01	6.66 *E*−03	3.97 *E*−02
			−	+	+	=	+

DTLZ3	Ave	**1.82* E*** **+** **02**	1.88 *E* + 02	1.92 *E* + 02	5.19 *E* + 02	1.89 *E* + 02	2.08 *E* + 02
	Std	**1.09* E* + 01**	1.13 *E* + 01	3.58 *E* + 01	6.96 *E* + 01	1.15 *E* + 01	2.22 *E* + 01
			+	+	+	+	+

DTLZ4	Ave	**7.94* E*−02**	8.54 *E*−02	2.10 *E*−01	5.53 *E*−01	9.54 *E*−02	8.08 *E*−02
	Std	**1.01* E*−02**	1.09 *E*−02	6.10 *E*−02	6.27 *E*−02	2.48 *E*−02	2.40 *E*−02
			+	+	+	+	=

DTLZ5	Ave	2.28 *E*−02	2.23 *E−*02	6.59 *E*−02	3.81 *E*−01	3.76 *E*−02	**2.07* E*−02**
	Std	**3.66* E*−03**	3.73 *E*−03	1.83 *E*−02	4.35 *E*−02	5.94 *E*−03	4.56 *E*−03
			=	+	+	+	=

DTLZ6	Ave	1.66 *E*−02	**8.54* E*−03**	1.17 *E*−01	8.42 *E + *00	1.72 *E*−02	8.73 *E*−03
	Std	1.41 *E*−03	**6.39* E*−04**	8.50 *E*−02	1.20 *E*−01	4.83 *E*−03	6.96 *E*−04
			−	+	+	=	−

DTLZ7	Ave	**8.90* E*−02**	1.12 *E*−01	1.28 *E *+ 00	8.70 *E + *00	8.98 *E*−02	4.89 *E*−01
	Std	**6.25* E*−03**	5.41 *E*−02	1.81 *E*−01	7.83 *E*−01	6.91 *E*−03	2.08 *E*−01
			+	+	+	=	+
	*w*\*t*\*l*		7\1\2	9\1\0	9\0\1	7\3\0	6\2\2

**Table 5:** Results of the IGD for the structural optimization problem.

IGD	DMOEOA	MOPSO	MOALO	NSGAII	MOWOA	MOGWO
Best	**1.02* E* + 01**	1.14 *E* + 01	2.29 *E* + 01	5.41 *E* + 01	1.12 *E* + 01	1.07 *E* + 01
Worst	1.75 *E* + 01	**1.62* E* + 01**	1.39 *E* + 02	1.92 *E* + 02	1.92 *E* + 01	2.30 *E* + 01
Average	**1.27* E* + 01**	1.29 *E* + 01	3.95 *E* + 01	1.12 *E* + 02	1.37 *E* + 01	1.56 *E* + 01
Std.	**1.38* E* + 00**	1.41 *E* + 00	2.20 *E* + 01	3.64 *E* + 01	2.01 *E* + 00	2.66 *E* + 00

**Table 6:** Nomenclature table for variables and parameters.

*X*	Decision vector
*F*(*X*)	Objective vector
GL(*X*)	Grid location
GR(*X*)	Grid ranking
GCPD(*X*)	Grid coordinate point distance
*C*	Concentration of an individual
*C*_*e*_	Equilibrium candidate
*C*_*e*,pool_	Equilibrium pool
*Q*	Disruption coefficient
*F*	Exponential term
*G*	Generation rate
Pop	Population
Rep	External repository
Δ	Metric of delta
IGD	Inverted generational distance
div	Number of grid divisions
*d*	Width of the grid
*V*	Unit
*a*1	Exploration parameter
*a*2	Exploitation parameter
GP	Generation probability
*κ*	Decay vector
IT	Max iteration
*K*	Number of objectives
*N*	Size of population
*R*	Size of external repository

## Data Availability

All data included in this study are available from the corresponding author upon request.
